# Switching Design for Assessment of Interchangeability in Biosimilar Studies

**DOI:** 10.3390/pharmaceutics18020187

**Published:** 2026-01-31

**Authors:** Yuqing Liu, Wendy Lou, Shein-Chung Chow

**Affiliations:** 1 Department of Biostatistics, Dalla Lana School of Public Health, University of Toronto, Toronto, ON M5T 3M7, Canada; wendy.lou@utoronto.ca; 2 Department of Biostatistics and Bioinformatics, School of Medicine, Duke University, Durham, NC 27705, USA; sheinchung.chow@duke.edu

**Keywords:** biosimilars, drug interchangeability, switching design, alternating treatment designs, balanced designs, carryover effect, N-of-1 trial design, intra-subject variability

## Abstract

**Background:** In biosimilar studies, assessing the switchability and interchangeability of biosimilars with their reference products is essential for ensuring reliable clinical evaluation. This study explores optimal trial design strategies incorporating balanced and uniform structures to enhance statistical efficiency in treatment effect under a carryover setting. **Methods:** Using a linear mixed-effect model for log-transformed responses, we conducted a theoretical variance-based evaluation of all possible two-treatment switching designs in three-period and four-period crossover trials, considering settings with and without carryover effects. A total of 247 distinct three-period designs and 65,519 distinct four-period designs were enumerated and classified according to structural properties, with particular attention to those incorporating a non-switching arm (NSA). **Results:** SBUwP-NSA (Strongly Balanced Uniform-within-Period designs with a Non-Switching Arm) consistently achieved the minimum variance for treatment effect estimation in both carryover and no-carryover settings. In the absence of carryover effects, UwP-NSA (Uniform-within-Period designs with a Non-Switching Arm) attained equivalent efficiency. In contrast, commonly used dedicated switching designs exhibited substantially lower relative efficiency, achieving as little as 50–55% of the efficiency of the optimal designs, depending on carryover assumptions. **Conclusions:** This comprehensive theoretical evaluation demonstrates that incorporating strong balance and uniformity properties can yield substantial efficiency gains in switching studies. The results provide quantitative guidance for selecting efficient crossover designs, enabling improved estimation precision while maintaining practical relevance for interchangeability and switching assessments in biosimilar research.

## 1. Introduction

The assessment and use of biosimilars have become an essential component of modern drug development due to their great potential to reduce healthcare costs and expand access to biological therapies [1,2]. A key consideration in biosimilar development is the interchangeability of a biosimilar with its reference product, meaning the biosimilar can be substituted for the reference without intervention from the prescriber and without increased risk or diminished efficacy [3,4,5]. In the United States, interchangeability is a formal regulatory designation that requires evidence from a switching study demonstrating that alternating between the biosimilar and reference does not adversely affect safety or effectiveness [6,7]. In contrast, agencies such as the European Medicines Agency (EMA) and the World Health Organization (WHO) generally interpret interchangeability as clinical equivalence when switching, considering approved biosimilars as therapeutic alternatives to their reference products without requiring a separate regulatory designation [8,9,10]. Similarly, Health Canada considers authorized biosimilars to be clinically interchangeable with their reference biologics, leaving substitution policies to provincial or territorial authorities [11,12,13].

Specifically, one or more switching studies are usually used to administer biologics multiple times to support and demonstrate the safety or efficacy of alternating or switching between the biosimilars and their reference product. However, achieving this goal highly depends on the trial designs, which directly affect the accuracy of evaluating the treatment effects. The U.S. Food and Drug Administration (FDA) guidance [6,14] recommends a specific crossover trial design called ‘dedicated switching design’ as a general-principle suggestion of minimum criteria for interchangeability studies. Such a design typically involves an initial lead-in period on the reference product, followed by a randomized two-arm phase: one arm undergoes at least three switches between reference and biosimilar (with the final switch to the biosimilar), and the other arm remains on the reference product without any switches. This design ensures that both arms include exposure to the reference product, making it possible to directly compare outcomes between switching and non-switching strategies. While this two-sequence, four-period design (e.g., in a two-treatment setting where R denotes the reference product and T denotes the test product, with the sequences R → R → R → R and R → T → R → T) satisfies the minimum requirements for a direct comparison, it is unbalanced with respect to treatment allocation, as only one sequence receives the biosimilar. Under certain circumstances, this may lead to reduced statistical efficiency for estimating treatment and carryover effects.

To address these limitations, alternative crossover designs with improved balanced strategies have been proposed as better options for interchangeability, such as a complete N-of-1 trial and parallel-crossover hybrid design [15,16]. To find the optimal design, the strongly balanced-uniformity property was considered, which has been shown to be universally optimal for effect estimation in repeated measurement designs by ensuring every treatment appears equally in each period and that every treatment sequence transition occurs with equal frequency [17,18]. In the context of interchangeability, this concept could be reformed as Strongly Balanced Uniform-within-Period (SBUwP), which seeks to equalize the distribution of treatments across periods and the frequency of all transition types (including repeated same-treatment transitions) while keeping the ability to satisfy regulatory requirements by providing a non-switching arm as control for switching effects.

In this study, we conduct a comprehensive theoretical evaluation of two-treatment switching trial designs under three-period and four-period crossover settings to identify designs that optimize statistical efficiency for interchangeability assessment. We enumerate all possible treatment sequences and combinations (including those with and without a dedicated non-switching arm) and categorize them by their balance properties. The performance of each design is assessed in three key estimation contexts: (1) treatment effect estimation with carryover effects (reflecting a conservative scenario where residual effects from the previous period may bias outcomes); (2) treatment effect estimation without carryover (assuming any carryover is negligible, e.g., with sufficient washout between periods). Our goal is to determine which designs minimize the variance of these estimates, as lower variance corresponds to higher precision and power for a given sample size. By evaluating thousands of potential designs and comparing their variance characteristics, we aim to provide evidence-based guidance for selecting efficient and reliable trial designs for interchangeability studies.

The remainder of this paper is organized as follows. Section 2 describes the methodological framework, including the study design, statistical modeling, and evaluation of design performance. Section 3 presents the main results, comparing estimator efficiency metrics across different switching design structures. Section 4 discusses the implications of the findings, highlighting practical considerations for study implementation and potential extensions for future research.

## 2. Methods

An interchangeable biosimilar is one that can be substituted for its reference product without requiring intervention from the prescribing healthcare provider [3,6]. This designation not only requires the biosimilar to be highly similar to the reference product in terms of safety, efficacy, and quality, but also demands additional evidence demonstrating that alternating or switching between the products does not increase risk or diminish efficacy [6]. To meet regulatory expectations, dedicated switching studies recommended by the FDA are typically used in interchangeability assessment as a minimum criterion to evaluate the potential impact of switching.

In bioequivalence (BE) and biosimilarity (BS) studies, design balance is essential to achieve the highest possible efficiency [19], where a balanced design is defined by the following criteria: 1. all participants received each of treatments once within each sequence, 2. each treatment appears an equal number of times across study periods, and 3. the number of participants switching from one treatment to another treatment (e.g., T → R and R → T) in the adjacent period is the same for all treatment pairs. Achieving balance provides a consistent framework for accurately comparing treatments, ensuring that the results are not influenced by imbalances in treatment allocation. For the case with more periods, the SBU (Strongly Balanced Uniform) design, originally developed in the theory of RMD (repeated-measurement design), has been proven to exhibit optimality by ensuring equal representation of all elements, thereby optimizing statistical efficiency and minimizing bias [17]. The SBU design refers to the following: 1. in the order of treatment assignment, each treatment is preceded by every other treatment (including itself) the same number of times; 2. uniform within periods, where an equal number of sequences are assigned to each treatment in each period; 3. uniform within sequences, where each treatment appears the same number of times in each sequence. Such a design ensures equal representation of all treatments, thereby optimizing statistical efficiency and providing robust inference for treatment and carryover effects.

However, in the context of interchangeability assessment, SBU designs are not always feasible or applicable. In practice, there is limited research comparing feasible switching designs to identify optimal configurations for multiple switches while considering study practicalities, such as the impact of the number of periods and sequences on efficiency. Moreover, the commonly used switching design recommended by the FDA involves a non-switching arm [6], which violates the first and third constraints of SBU design due to repeated exposures to the same product in a single sequence, which could result in unchanged transitions (e.g., R → R). Furthermore, the switching design also failed to meet the criterion of uniformity within sequences for Strongly Balanced Uniform design due to regulatory requirements for non-switching arms [6]. To identify the optimal switching design that incorporates a non-switching arm, this research examines the Strongly Balanced Uniform-within-Period Switching Design with Non-Switching Arm (SBUwP-NSA), which allows for a fair comparison between the switching and non-switching arms, addressing key regulatory requirements and ensuring reliable and consistent results. Uniformity within a period ensures that each treatment appears the same number of times across all periods, and being strongly balanced ensures that all treatment transition pairs (e.g., T → R, R → T, T → T, R → R) occur with equal frequency.

To evaluate the statistical efficiency and operating characteristics of alternative crossover switching designs for assessing the interchangeability of two biosimilars, designs were compared based on three core criteria: statistical efficiency, power, and operational feasibility, under a fixed total sample size

In this research, optimality is defined as the minimization of within-subject variability, which includes drug effects, period effects, and carryover effects, while treating sequence effects as part of between-subject variability [20,21]. Designs that reduce this variability are considered superior, as they enhance the precision of treatment effect and carryover effect estimations, thereby improving the reliability and robustness of statistical analyses in interchangeability assessments. By integrating balancing and uniformity properties into the switching design, we aim to enhance the assessment process and improve the precision of statistical analyses in interchangeability evaluations.

### 2.1. Study Design

In this study, we evaluated all possible drug regimens under a J-period setting for M-drug scenarios. Each design was characterized by its number of periods (J), number of sequences (K), and the structure properties of treatment allocation across periods and sequences. Specifically, the complete N-of-1 design framework, which includes $K=M^{J}$ distinct sequences, was explored. For each number of feasible sequences k=2, …, K, $\left(\begin{array}{c} K\\ k \end{array}\right)$ combinations of k sequence designs were created, resulting in a total of $2^{K}-1-K$ designs. Therefore, for a K-sequence J-period design, there are a total of (J−1)K transitions between adjacent drug administrations. To achieve uniformity within periods, the number of sequences K must be a multiple of the number of drugs M, i.e., M|K. Additionally, to ensure an equal number of transitions, the total number of transitions must be a multiple of $M^{2}$, the number of types in transition of adjacent drug administration [17], i.e., $M^{2}|(J-1)K$.

For easy illustration, we focus on designs involving M=2 treatment, the test product (T) and the reference formulation (R), administered in J=3 and J=4 periods, settings commonly used in bioequivalence and biosimilarity assessments. The enumeration resulted in 247 three-period designs and 65,519 four-period designs, cataloged by structural properties in Table A1 and Table A2. Within each configuration, designs were classified according to structural properties, including balance, allocation symmetry, and uniformity:

1. Strongly Balanced (SB): Ensures adjacency balance, such that forward and reverse transitions (T → R and R → T) occur with equal frequency.
2. Uniform-within-Period (UwP): Ensures equal treatment frequencies within each period but not necessarily across sequences.
3. Uniform-within-Sequence (UwS): Ensures that each sequence contains an equal number of each treatment, though period-wise balance is not guaranteed.

In addition, combined structures, including Strongly Balanced and Uniform (SBU), SB with Period Uniformity (SBUwP), SB with Sequence Uniformity (SBUwS), and Uniform (UwP + UwS) designs, were generated to capture intermediate designs, along with unbalanced, non-uniform (None) designs for reference. These classifications allow systematic evaluation of how structural properties influence the variance of treatment effect estimation and statistical power, particularly when switching patterns increase in complexity. Then, for each enumerated design, the fixed-effects design matrix (X) was constructed and used to compute the variance of the estimated treatment contrast under the mixed-effects model (described in Section 2.2). Designs were subsequently ranked by estimator variance and relative efficiency, defined relative to the minimum-variance design for an equal total sample size.

### 2.2. Statistical Methodology

The average bioequivalence approach is the fundamental statistical method used to demonstrate interchangeability of the proposed product in switching studies, where the results of switching between the biosimilar and reference product are neither significantly worse nor better than those of the reference biologic.

#### 2.2.1. Hypothesis

The assessment of PK (pharmacokinetics) parameters typically involves calculating $\left(1-2\alpha \right)\times 100\%$ (e.g., 90%) confidence intervals for the ratio of the geometric means of the biosimilar and reference product responses. Under the assumption that the log-transformation could remove the skewness of responses ${\tilde{Y}}_{ijk}$ and log-transformed data $Y_{ijk}$ are approximately normally distributed, the point estimate and the confidence intervals on the original scale can be obtained by back-transformation (i.e., antilog) of the corresponding estimate and interval on the logarithmic scale. If the confidence interval falls within the predefined equivalence margins, bioequivalence is concluded.

In general, the 80/125 rule is a commonly used standard required by most regulatory agencies for average bioavailabilities based on the average bioequivalence method. This rule uses (80%,125%) as a bioequivalence limit for the confidence interval for the ratio of average bioavailabilities, which is symmetric to 0 on the log-scale. For the additive raw data model, the hypotheses can be expressed as

$$H_{0}:\;{\tilde{\theta }}_{mm^{\prime }}\leq {\tilde{\theta }}_{L}\;or\;\tilde{\theta }\geq {\tilde{\theta }}_{U}\;vs.\;H_{a}:\;{\tilde{\theta }}_{L}<{\tilde{\theta }}_{mm^{\prime }}<{\tilde{\theta }}_{U}$$

where ${\tilde{\theta }}_{mm^{\prime }}={\tilde{\mu }}_{m}/{\tilde{\mu }}_{m^{\prime }}$ is the ratio of the median bioavailabilities of the mth and $m^{\prime }$th formulations [22], and $\left({\tilde{\theta }}_{L},{\tilde{\theta }}_{U}\right)=(0.80,1.25)$ denotes the bioequivalent limits. In a logarithmic scale multiplicative model, the parameter of interest becomes $\theta_{mm^{\prime }}=\log{\tilde{\theta }}_{mm^{\prime }}=\log{\tilde{\mu }}_{m}/{\tilde{\mu }}_{m^{\prime }}=\mu_{m}-\mu_{m^{\prime }}$, and the null hypothesis becomes

$$H_{0}:\theta_{mm^{\prime }}\leq \theta_{L}\;or\;\theta_{mm^{\prime }}\geq \theta_{U}\;vs.\;H_{1}:\theta_{L}<\theta_{mm^{\prime }}<\theta_{U}$$

where $\left(\theta_{L},\theta_{U}\right)=(\ln{\tilde{\theta }}_{L},\ln{\tilde{\theta }}_{U})=\left(\ln0.8,\ln1.25\right)$ and $\theta_{L}=-\theta_{U}$.

#### 2.2.2. Statistical Model

To assess the interchangeability of test product (T) and reference formulation (R) under a K-sequence by J-period (denoted by K×J) design, let ${\tilde{Y}}_{ijk}$ be the response of the ith subject in the kth sequence at the jth period; the generalized multiplicative model [22] can be expressed as

$${\tilde{Y}}_{ijk}=\tilde{\mu }{\tilde{S}}_{ik}{\tilde{P}}_{j}{\tilde{F}}_{d\left(j,k\right)}{\tilde{C}}_{d\left(j-1,k\right)}{\tilde{e}}_{ijk}$$

where $i=1,\ldots ,n_{k},j=1,\ldots ,J,k=1,\ldots ,K$; $n_{k}$ is the number of subjects in the kth sequence, and the total sample size $N=\sum_{k=1}^{K}n_{k}$. Then, the equivalent log-transformed model that meets the assumptions of normality and homogeneity of variances can be described by

$$Y_{ijk}=\mu +S_{ik}+P_{j}+F_{d\left(j,k\right)}+C_{d(j-1,k)}+e_{ijk}$$

where

μ is the overall mean;

$S_{ik}$ is the random effect of the ith subject in the kth sequence with $S_{ik}\sim_{i.i.d.}N(0,\sigma_{S}^{2})$;

$P_{j}$ is the fixed effect of the jth period, where $\sum_{j}P_{j}=0$; $d\left(j,k\right)$ is the formulation in the kth sequence which is administered at the jth period.

$F_{d\left(j,k\right)}$ is the fixed drug effect of $d\left(j,k\right)$, the formulation administered in the kth sequence at the jth period, where $d\left(j,k\right)\in treatment\;set\{1,\ldots ,M\}$ and $\sum_{m=1}^{M}F_{m}=0$.

$C_{d(j-1,k)}$ is the fixed first-order carryover effect of the formulation in the kth sequence which is administered at the (j−1)th period, where $C_{d(0,k)}=0$ and $\sum_{m=1}^{M}C_{m}=0$.

$e_{ijk}$ is the within-subject random error with $e_{ijk}\sim_{i.i.d.}N(0,\sigma_{e}^{2})$, where $\sigma_{e}^{2}$ denotes the variance of the intra-subject residual of log-transformed measures with v degrees of freedom.

In this research, the statistical analysis was conducted under the assumptions of the mutual independence of inter-subject variability and intra-subject variability [22] and the same variance for all formulations $\sigma_{T}^{2}=\sigma_{R}^{2}=\sigma_{e}^{2}$. In addition, to ensure the design has sufficient observations to adequately model J period effects, M treatment effects, and M carryover effects, the number of sequences in the design must satisfy the following criterion of the overall degrees of freedom (df): df=JK−1≥2(M−1)+(J−1). Therefore, at least 2 sequences are needed in both 3-period design and 4-period design to assess interchangeability of 2 treatments.

#### 2.2.3. Effect Estimations

Using this additive linear model, the performance of each design was assessed in three estimation contexts: 1. Treatment effect estimation with the presence of unequal carryover effects. 2. Treatment effect estimation without carryover effects. 3. Carryover effect estimation.

Let ${\bar{Y}}_{\cdot jk}=\frac{1}{n_{k}}\sum_{i=1}^{n_{k}}Y_{ijk}$ be the observation mean at the kth sequence and jth period, the vector of observed sequence-by-period means is then denoted as $\bar{Y}={\left({\bar{Y}}_{\cdot 11},{\bar{Y}}_{\cdot 12},\ldots ,{\bar{Y}}_{\cdot JK}\right)}^{\prime }$. Based on the model, let P be the parameter vector containing all unknown parameters in the model with length 1+J+2M, X be the design matrix of model parameters, and L be the linear contrast to denote the parameter of interest δ as $L^{\prime }P$. According to Chow and Liu [22], the unbiased estimator of the parameter of interest can be obtained by $\hat{\delta }=\beta^{\prime }\bar{Y}$, where $\beta ={\left(X^{\prime }X\right)}^{-1}X^{\prime }L$ is the coefficient vector. For a design matrix with a singular Gram matrix $X^{\prime }X$, the generalized inverse was calculated to obtain the coefficient vector.

Convert the estimator into matrix format, and then it can be expressed by $\hat{\delta }=\sum_{k=1}^{K}\sum_{j=1}^{J}C_{jk}{\bar{Y}}_{\cdot jk}$, where $C_{jk}$ denote the coefficients of the linear contrasts of sequence-by-period means, which correspond to the $\left(\left(j-1\right)K+k\right)$th coefficients in β. Meanwhile, the variance of the estimator can be derived by $Var\left(\hat{\delta }\right)=\sigma_{e}^{2}\sum_{k=1}^{K}\frac{1}{n_{k}}\sum_{j=1}^{J}C_{jk}^{2}$ [22,23]. When $n_{1}=\ldots =n_{K}=n$, the total sample size becomes N=nK. Let $b=\sum_{k=1}^{K}\sum_{j=1}^{J}C_{jk}^{2}$ denote the sum of the squares of the coefficients of the linear contrasts of sequence-by-period means; a lower value means less variability in the contrast coefficients. Multiplying contrast weighting b by the number of sequences K, bK scales the contrast weighting across all sequences, accounting for the structure and size of the design [24]. Then, the variance of the three effect estimations can be expressed by $Var\left(\hat{\delta }\right)=\frac{\sigma_{e}^{2}}{n}\sum_{k=1}^{K}\sum_{j=1}^{J}C_{jk}^{2}=\sigma_{e}^{2}\frac{b}{n}=\sigma_{e}^{2}\frac{bK}{N}$, which serves as the primary metric for evaluating design efficiency in this research. The variance is directly proportional to bK while being inversely proportional to the total sample size N. In other words, with the same total sample size N, a lower bK indicates a lower variance and higher efficiency of the design.

#### 2.2.4. Software

We performed the symbolic matrix operation in exact fractional form using Python (version 3.13.1) [25]. The primary computational libraries included SymPy (version 1.13.3), NumPy (version 2.1.3) [26], pandas (version 2.2.3) [27], and Matplotlib (version 3.9.2) [28].

## 3. Results

This study evaluates the performance of trial designs in different estimation contexts under three-period and four-period settings, including variances of treatment effect estimation with and without carryover effects. Minimum variances for each estimation context are summarized in the following tables, demonstrating the efficiency of different designs. The results highlight the performance of different design structures and numbers of periods or sequences in minimizing variability.

### 3.1. Three-Period Designs

For three-period designs, only four types of design are present: SBUwP, UwP, SB, and None. Table 1 presents the minimum variance values observed under various three-period settings. It can be observed that, for designs with an even number of sequences, the SBUwP (Balanced Uniform-within-Period) designs and the UwP designs exhibit similar performance in scenarios without carryover effects. However, in the presence of carryover effects, SBUwP designs demonstrate superior performance by achieving lower variance compared to any other designs, while UwP is the second-best performing design strategy for treatment effect estimation. This highlights the advantage of SBUwP designs in scenarios where carryover effects cannot be ignored, ensuring more precise and reliable estimations of treatment effects. Notably, SBUwP designs achieve the same minimum variance across all sequence settings, reflecting their optimal structure for minimizing variability. The strongly balanced (SB) design only appears under a 4 × 3 setting, which has a smaller estimation variance compared to the None design when the carryover effect is present, but worse without the carryover effect.

For odd-sequence designs, there is only the None design shown due to the inherent constraints of odd-numbered sequences. Across all estimation settings, the variance for odd-sequence designs decreases as the number of sequences increases, indicating improved statistical efficiency with higher sequence numbers. However, the variances of these None designs for all sequence settings are consistently larger than the minimum variance achieved by SBUwP designs.

Additionally, with the exception of the 2 × 3 design, which exhibited notable limitations due to its structural imbalance, all evaluated designs incorporating a non-switching arm (NSA) were capable of achieving the minimum variance under the same settings. These designs demonstrate strong performance in minimizing treatment effect estimation variance and can potentially be utilized in interchangeability assessments. The inclusion of a non-switching arm in these designs provides a robust comparison framework while maintaining statistical efficiency, making them suitable for regulatory evaluations of biosimilar interchangeability. Additionally, five SBUwP-NSA structures mentioned above achieve minimum variance in both with- and without-carryover-effects scenarios: two four-sequence designs (i.e., TTT/TRT/RTR/RRR, TTR/TRT/RTT/RRR), two six-sequence designs (i.e., TTT/TTR/TRT/RTR/RRT/RRR, TTT/TRT/TRR/RTT/RTR/RRR), and an eight-sequence complete N-of-1 design (i.e., TTT/TTR/TRT/TRR/RTT/RTR/RRT/RRR). In contrast, four UwP-NSA designs achieve minimum variance only in the absence of carryover effects: A two-sequence design (i.e., TTT/RRR), two four-sequence designs (i.e., TTT/TTR/RRT/RRR, TTT/TRR/RTT/RRR), and a six-sequence design (i.e., TTT/TTR/TRR/RTT/RRT/RRR). While these designs do not satisfy equal frequency of transition pairs (and thus are not strongly balanced for carryover), they do maintain within-period uniformity, supporting efficient treatment effect estimation when carryover is negligible.

The two-treatment, three-period results demonstrated that SBUwP (Strongly Balanced Uniform-within-Period) designs consistently achieved the smallest variance across all settings, highlighting their superior performance in scenarios with carryover effects. For even-sequence designs, Non-SBUwP designs achieved comparable minimum variance to SBUwP designs for treatment effect estimation with carryover effect estimation, except for the 2 × 3 design, which displayed limitations. In contrast, odd-sequence designs that failed to meet the SBUwP criteria demonstrated higher variance, though the variance decreased with an increasing number of sequences.

The inclusion of non-switching arms, particularly in SBUwP-NSA designs, demonstrated the potential to maintain efficiency while providing robust statistical evaluations. These findings highlight the importance of strongly balancing and uniformity in switching designs for improving the precision of treatment and carryover effect estimations. The identified designs provide practical and efficient frameworks for interchangeability assessments, offering valuable insights for regulatory evaluations of biosimilar products.

### 3.2. Four-Period Designs

Findings from the four-period setting, summarized in Table 2, further reinforce the patterns observed in three-period designs. A total of seven types of designs are shown; SBUwP (Strongly Balanced Uniform-within-Period) designs again achieved the same lowest variance across all estimation contexts as the SBU design under the 4 × 4 setting, confirming their optimality in drug effect estimation. Without the carryover effect, UwP and Uniform designs also demonstrated equivalent performance to SBUwP and SBU designs in treatment effect estimation. However, with the consideration of non-zero carryover effects, the estimated variance of the UwP and Uniform designs increased slightly, whereas UwS and SB designs generated larger variance, and even exceeded that of the None design under some scenarios. In contrast, odd-numbered sequence designs continued to exhibit a reduction in variance as the number of sequences increased; however, their efficiency remained lower compared to SBUwP designs and UwP designs.

Especially for the two-sequence, four-period (2 × 4) setting, which includes the minimum FDA-recommended dedicated switching framework (i.e., RTRT/RRRR), the variance factor bK = 2 is observed both with and without carryover effects. This performance is comparable to that of UwS designs but remains less efficient than designs possessing Uniform and UwP properties, achieving 54.5% and 50.0% relative efficiency when carryover effects are present and absent, respectively, and only 66.7% of the most efficient designs within the None class. These results indicate that, even within minimum regulatory configurations, incorporating uniformity properties yields measurable gains in estimation efficiency.

Notably, all four-period designs that achieved the minimum variance incorporated a non-switching arm, shown in Table A3, underscoring its value in enhancing design robustness. In addition, it can be seen that under the assumption of the normality and homogeneity of variances, all evaluated SBUwP-NSA designs achieved the same minimum variance as the classic SBU design (i.e., TTRR/TRTR/RTRT/RRTT) in both the presence and absence of carryover effects. These results highlight the potential methodological advantage of incorporating a non-switching arm in switching designs, as it allows for precise and robust evaluation of treatment and carryover effects while maintaining statistical efficiency, where minimizing variability and ensuring estimation accuracy are critical for regulatory and clinical decision-making.

### 3.3. Findings

In total, the current results demonstrated that the Strongly Balanced Uniform (SBU) design and Strongly Balanced Uniform-within-Period (SBUwP) designs consistently achieved the minimum variance across all estimation contexts, demonstrating their robustness and efficiency, particularly in scenarios with carryover effects, highlighting their superior statistical performance. Among even-sequence designs, several UwP and Uniform configurations achieved variance levels comparable to SBUwP designs for drug effect estimation without carryover effects, making them viable alternatives when carryover effects can be assumed negligible, such as with sufficient washout periods. In contrast, None designs that did not meet any strongly balanced or uniformity criteria, while showing reduced variance with increasing sequence numbers, consistently exhibited higher estimation variance compared to SBU, SBUwP, and UwP designs, highlighting their relative inefficiency. Importantly, in both three-period and four-period settings, all designs achieving the minimum variance, except for the 2 × 3 SBUwP design and 4 × 4 SBU design, included configurations with a non-switching arm. Therefore, for interchangeability assessments, SBUwP designs with non-switching arms are recommended for treatment effect estimation, especially in the presence of carryover effects. In scenarios where carryover effects are ignorable, UwP designs with non-switching arms can also be effectively utilized. These findings emphasize the critical role of balanced and uniform designs in achieving robust and precise statistical evaluations.

## 4. Discussion

This study systematically evaluated all possible two-treatment switching designs under three-period and four-period settings to identify configurations that minimize estimation variance and are suitable for interchangeability assessments. Specifically, the evaluation focused on design efficiency under crossover model assumptions, with the aim of selecting optimal designs instead of prescribing a single universally sufficient design.

The results of this study provide valuable insights into the optimal design of switching studies for interchangeability assessments. SBUwP designs effectively reduce within-subject variability by ensuring uniform treatment allocation and equal transitions across study periods, making them highly suitable for scenarios involving carryover effects. UwP designs emerged as viable alternatives in settings without carryover effects but were less efficient in their presence. This highlights the necessity of strong balance and uniformity in designs where carryover effects are a concern, a situation frequently encountered in biologic switching studies. Conversely, UwS, SB, and None designs demonstrated higher variance, even as variance decreased with more sequences. These designs, while improving with increasing sequence numbers, remain suboptimal compared to their even-numbered counterparts. The inclusion of non-switching arms in SBUwP designs that achieved the minimum variance (except for the 2 × 3 design) reflects their critical role in interchangeability assessments. Non-switching arms serve as controls that stabilize estimation of treatment and carryover effects, thereby improving interpretability while maintaining statistical precision.

However, several limitations remain. The analysis focuses primarily on three-period and four-period settings with two treatments, which may not fully capture the complexities of longer study durations or multi-treatment scenarios. Additionally, while the study emphasizes within-subject variability, the potential impact of between-subject variability, such as individual differences, is not explicitly addressed. Such variability can influence design efficiency, required sample size, study duration, and operational cost in real-world biosimilarity studies. Another limitation is the reliance on theoretical assumptions regarding homoscedasticity, which may not always align with real-world conditions. Accordingly, cost considerations and operational constraints were not formally modeled and are acknowledged as practical limitations of the present work. Future research should explore the scalability of these findings to more complex designs with extended periods and additional treatments. Investigations into the interaction of within- and between-subject variability, as well as sensitivity analyses under varying assumptions of carryover effects, are also warranted. Furthermore, simulation-based evaluations informed by realistic trial settings are an important next step and are currently being pursued to bridge theoretical efficiency and practical feasibility.

Notably, regulatory perspectives on interchangeability studies are evolving. In 2024, the FDA issued draft guidance indicating that additional switching studies will generally not be required for biosimilar interchangeability approval [29], reflecting accumulating scientific evidence that switching between a reference biologic and its biosimilar does not meaningfully increase risks or affect efficacy. More recently, updated FDA draft guidance further emphasizes that comparative analytical assessments, supported by pharmacokinetic and immunogenicity evaluations, are often more sensitive than comparative clinical efficacy studies for detecting clinically meaningful differences, and that such efficacy studies may not be necessary when residual uncertainty has been adequately addressed under a totality-of-the-evidence framework [30]. Importantly, these guidelines emphasize minimum evidentiary criteria rather than asserting that any single study design is universally sufficient. However, the regulatory expectations continue to differ across international jurisdictions, where switching and interchangeability evidence may still be relevant. Beyond regulatory decision-making, the scientific question of whether switching affects clinical outcomes remains relevant for clinicians, patients, and researchers. Our study contributes to this broader context by providing efficient design strategies for evaluating switching effects. Even with regulatory changes, switching studies may remain valuable in other contexts, such as clinical practice evaluations, single-patient (N-of-1) trials, and post-marketing studies aimed at informing real-world use.

Overall, these findings emphasize the need to prioritize SBU and SBUwP designs and the consideration of the inclusion of non-switching arms in switching studies. By addressing both regulatory requirements and statistical efficiency, the identified designs offer a practical foundation for reliable and efficient interchangeability evaluations. Future work will focus on extending these findings to real-world applications through simulation and applied case studies, as well as to more complex treatment scenarios involving additional treatments and longer study durations.

## Figures and Tables

**Table 1:** Minimum bK ^1^ of 3-period treatment effect estimations by design structures.

Design K × J	Treatment Effect Estimation Setting	SBUwP ^2^	UwP	SB	None
2 × 3	with Carryover	4/3	4	-	2
	without Carryover	4/3	4/3	-	2
3 × 3	with Carryover	-	-	-	3/2
	without Carryover	-	-	-	3/2
4 × 3	with Carryover	4/3	8/5	48/25	16/11
	without Carryover	4/3	4/3	16/9	16/11
5 × 3	with Carryover	-	-	-	25/18
	without Carryover	-	-	-	25/18
6 × 3	with Carryover	4/3	36/25	-	18/13
	without Carryover	4/3	4/3	-	18/13
7 × 3	with Carryover	-	-	-	49/36
	without Carryover	-	-	-	49/36
8 × 3	with Carryover	4/3	-	-	-
	without Carryover	4/3	-	-	-

^1^ bK is a measure of the design’s structural contribution to variance, where $Var\left(\hat{\theta }\right)=\frac{bK}{N}\sigma_{e}^{2}$ and $b=\sum_{k=1}^{K}\sum_{j=1}^{J}C_{jk}^{2}$. ^2^ None of the SBUwP 2 × 3 designs have a non-switching arm.

**Table 2:** Minimum bK ^1^ of 4-period treatment effect estimations by design structures.

Design K × J	Estimation Setting	SBU	Uniform	SBUwP	UwP	UwS	SB	None
2 × 4	with Carryover	-	12/11	-	12/11	2	-	4/3
	without Carryover	-	1	-	1	2	-	4/3
3 × 4	with Carryover	-	-	-	-	9/8	-	9/8
	without Carryover	-	-	-	-	9/8	-	9/8
4 × 4	with Carryover	1	12/11	1	12/11	176/153	160/139	16/15
	without Carryover	1	1	1	1	8/7	8/7	16/15
5 × 4	with Carryover	-	-	-	-	50/47	-	450/431
	without Carryover	-	-	-	-	25/24	-	25/24
6 × 4	with Carryover	-	12/11	-	108/107	-	-	36/35
	without Carryover	-	1	-	1	-	-	36/35
7 × 4	with Carryover	-	-	-	-	-	-	1764/1727
	without Carryover	-	-	-	-	-	-	49/48
8 × 4	with Carryover	-	-	1	48/47	-	2944/2851	64/63
	without Carryover	-	-	1	1	-	32/31	64/63
9 × 4	with Carryover	-	-	-	-	-	-	1215/1199
	without Carryover	-	-	-	-	-	-	81/80
10 × 4	with Carryover	-	-	-	300/299	-	-	100/99
	without Carryover	-	-	-	1	-	-	100/99
11 × 4	with Carryover	-	-	-	-	-	-	5445/5398
	without Carryover	-	-	-	-	-	-	121/120
12 × 4	with Carryover	-	-	1	108/107	-	5088/5017	144/143
	without Carryover	-	-	1	1	-	78/71	144/143
13 × 4	with Carryover	-	-	-	-	-	-	2366/2351
	without Carryover	-	-	-	-	-	-	169/168
14 × 4	with Carryover	-	-	-	588/587	-	-	196/195
	without Carryover	-	-	-	1	-	-	196/195
15 × 4	with Carryover	-	-	-	-	-	-	4725/4702
	without Carryover	-	-	-	-	-	-	225/224
16 × 4	with Carryover	-	-	1	-	-	-	-
	without Carryover	-	-	1	-	-	-	-

^1^ bK is a measure of the design’s structural contribution to variance, where $Var\left(\hat{\theta }\right)=\frac{bK}{N}\sigma_{e}^{2}$ and $b=\sum_{k=1}^{K}\sum_{j=1}^{J}C_{jk}^{2}$.

## Data Availability

The original contributions presented in this study are included in the article. Further inquiries can be directed to the corresponding author.

## Appendix A

**Table A1:** Summary table of 3-period designs by three structures of treatment regimen.

3-Period Design (247 Designs)
**SBUwP Designs (9 designs), including 5 NSA designs** 2-sequence (2 designs without non-switching arm): TTR/RRT, TRR/RTT 4-sequence (4 designs, 2 with non-switching arm): TTT/TRT/RTR/RRR, TTT/TRR/RTR/RRT, TTR/TRT/RTT/RRR, TTR/TRR/RTT/RRT, 6-sequence (2 designs with non-switching arm): TTT/TTR/TRT/RTR/RRT/RRR, TTT/TRT/TRR/RTT/RTR/RRR, 8-sequence (1 design with non-switching arm): TTT/TTR/TRT/TRR/RTT/RTR/RRT/RRR
**SB Designs (2 designs), including 1 NSA design** 4-sequence (2 designs, 1 with non-switching arm): TTT/TRT/TRR/RRT, TTR/RTT/RTR/RRR
**UwP Designs (8 designs), including 4 NSA designs** 2-sequence (2 designs, 1 with non-switching arm): TTT/RRR, TRT/RTR 4-sequence (4 designs, 2 with non-switching arm): TTT/TTR/RRT/RRR, TTT/TRR/RTT/RRR, TTR/TRT/RTR/RRT, TRT/TRR/RTT/RTR 2-sequence (2 design, 1 with non-switching arm): TTT/TTR/TRR/RTT/RRT/RRR, TTR/TRT/TRR/RTT/RTR/RRT
**None Designs (228 designs), including 117 NSA designs** 2-sequence (24 designs, 6 with non-switching arm): TTT/TTR, TTT/TRT, TTT/TRR, TTT/RTT, TTT/RTR, TTT/RRT, TTR/TRT, TTR/TRR, TTR/RTT, TTR/RTR, TTR/RRR, TRT/TRR, TRT/RTT, TRT/RRT, TRT/RRR, TRR/RTR, TRR/RRT, TRR/RRR, RTT/RTR, RTT/RRT, RTT/RRR, RTR/RRT, RTR/RRR, RRT/RRR 4-sequence (60 designs, 30 with non-switching arm): TTT/TTR/TRT/TRR, TTT/TTR/TRT/RTT, TTT/TTR/TRT/RTR, TTT/TTR/TRT/RRT, TTT/TTR/TRT/RRR, TTT/TTR/TRR/RTT, TTT/TTR/TRR/RTR, TTT/TTR/TRR/RRT, TTT/TTR/RTT/RTR, TTT/TTR/RTT/RRT, TTT/TTR/RTT/RRR, TTT/TTR/RTR/RRT, TTT/TTR/RTR/RRR, TTT/TTR/RRT/RRR, TTT/TRT/TRR/RTT, TTT/TRT/TRR/RTR, TTT/TRT/TRR/RRR, TTT/TRT/RTT/RTR, TTT/TRT/RTT/RRT, TTT/TRT/RTT/RRR, TTT/TRT/RTR/RRT, TTT/TRT/RRT/RRR, TTT/TRR/RTT/RTR, TTT/TRR/RTT/RRT, TTT/TRR/RTR/RRR, TTT/TRR/RRT/RRR, TTT/RTT/RTR/RRT, TTT/RTT/RTR/RRR, TTT/RTT/RRT/RRR, TTT/RTR/RRT/RRR, TTR/TRT/TRR/RTT, TTR/TRT/TRR/RTR, TTR/TRT/TRR/RRT, TTR/TRT/TRR/RRR, TTR/TRT/RTT/RTR, TTR/TRT/RTT/RRT, TTR/TRT/RTR/RRR, TTR/TRT/RRT/RRR, TTR/TRR/RTT/RTR, TTR/TRR/RTT/RRR, TTR/TRR/RTR/RRT, TTR/TRR/RTR/RRR, TTR/TRR/RRT/RRR, TTR/RTT/RTR/RRT, TTR/RTT/RRT/RRR, TTR/RTR/RRT/RRR, TRT/TRR/RTT/RRT, TRT/TRR/RTT/RRR, TRT/TRR/RTR/RRT, TRT/TRR/RTR/RRR, TRT/TRR/RRT/RRR, TRT/RTT/RTR/RRT, TRT/RTT/RTR/RRR, TRT/RTT/RRT/RRR, TRT/RTR/RRT/RRR, TRR/RTT/RTR/RRT, TRR/RTT/RTR/RRR, TRR/RTT/RRT/RRR, TRR/RTR/RRT/RRR, RTT/RTR/RRT/RRR 6-sequence (24 designs, 18 with non-switching arm): TTT/TTR/TRT/TRR/RTT/RTR, TTT/TTR/TRT/TRR/RTT/RRT, TTT/TTR/TRT/TRR/RTT/RRR, TTT/TTR/TRT/TRR/RTR/RRT, TTT/TTR/TRT/TRR/RTR/RRR, TTT/TTR/TRT/TRR/RRT/RRR, TTT/TTR/TRT/RTT/RTR/RRT, TTT/TTR/TRT/RTT/RTR/RRR, TTT/TTR/TRT/RTT/RRT/RRR, TTT/TTR/TRR/RTT/RTR/RRT, TTT/TTR/TRR/RTT/RTR/RRR, TTT/TTR/TRR/RTR/RRT/RRR, TTT/TTR/RTT/RTR/RRT/RRR, TTT/TRT/TRR/RTT/RTR/RRT, TTT/TRT/TRR/RTT/RRT/RRR, TTT/TRT/TRR/RTR/RRT/RRR, TTT/TRT/RTT/RTR/RRT/RRR, TTT/TRR/RTT/RTR/RRT/RRR, TTR/TRT/TRR/RTT/RTR/RRR, TTR/TRT/TRR/RTT/RRT/RRR, TTR/TRT/TRR/RTR/RRT/RRR, TTR/TRT/RTT/RTR/RRT/RRR, TTR/TRR/RTT/RTR/RRT/RRR, TRT/TRR/RTT/RTR/RRT/RRR
3-sequence (56 designs, 21 with non-switching arm): TTT/TTR/TRT, TTT/TTR/TRR, TTT/TTR/RTT, TTT/TTR/RTR, TTT/TTR/RRT, TTT/TTR/RRR, TTT/TRT/TRR, TTT/TRT/RTT, TTT/TRT/RTR, TTT/TRT/RRT, TTT/TRT/RRR, TTT/TRR/RTT, TTT/TRR/RTR, TTT/TRR/RRT, TTT/TRR/RRR, TTT/RTT/RTR, TTT/RTT/RRT, TTT/RTT/RRR, TTT/RTR/RRT, TTT/RTR/RRR, TTT/RRT/RRR, TTR/TRT/TRR, TTR/TRT/RTT, TTR/TRT/RTR, TTR/TRT/RRT, TTR/TRT/RRR, TTR/TRR/RTT, TTR/TRR/RTR, TTR/TRR/RRT, TTR/TRR/RRR, TTR/RTT/RTR, TTR/RTT/RRT, TTR/RTT/RRR, TTR/RTR/RRT, TTR/RTR/RRR, TTR/RRT/RRR, TRT/TRR/RTT, TRT/TRR/RTR, TRT/TRR/RRT, TRT/TRR/RRR, TRT/RTT/RTR, TRT/RTT/RRT, TRT/RTT/RRR, TRT/RTR/RRT, TRT/RTR/RRR, TRT/RRT/RRR, TRR/RTT/RTR, TRR/RTT/RRT, TRR/RTT/RRR, TRR/RTR/RRT, TRR/RTR/RRR, TRR/RRT/RRR, RTT/RTR/RRT, RTT/RTR/RRR, RTT/RRT/RRR, RTR/RRT/RRR 5-sequence (56 designs, 35 with non-switching arm): TTT/TTR/TRT/TRR/RTT, TTT/TTR/TRT/TRR/RTR, TTT/TTR/TRT/TRR/RRT, TTT/TTR/TRT/TRR/RRR, TTT/TTR/TRT/RTT/RTR, TTT/TTR/TRT/RTT/RRT, TTT/TTR/TRT/RTT/RRR, TTT/TTR/TRT/RTR/RRT, TTT/TTR/TRT/RTR/RRR, TTT/TTR/TRT/RRT/RRR, TTT/TTR/TRR/RTT/RTR, TTT/TTR/TRR/RTT/RRT, TTT/TTR/TRR/RTT/RRR, TTT/TTR/TRR/RTR/RRT, TTT/TTR/TRR/RTR/RRR, TTT/TTR/TRR/RRT/RRR, TTT/TTR/RTT/RTR/RRT, TTT/TTR/RTT/RTR/RRR, TTT/TTR/RTT/RRT/RRR, TTT/TTR/RTR/RRT/RRR, TTT/TRT/TRR/RTT/RTR, TTT/TRT/TRR/RTT/RRT, TTT/TRT/TRR/RTT/RRR, TTT/TRT/TRR/RTR/RRT, TTT/TRT/TRR/RTR/RRR, TTT/TRT/TRR/RRT/RRR, TTT/TRT/RTT/RTR/RRT, TTT/TRT/RTT/RTR/RRR, TTT/TRT/RTT/RRT/RRR, TTT/TRT/RTR/RRT/RRR, TTT/TRR/RTT/RTR/RRT, TTT/TRR/RTT/RTR/RRR, TTT/TRR/RTT/RRT/RRR, TTT/TRR/RTR/RRT/RRR, TTT/RTT/RTR/RRT/RRR, TTR/TRT/TRR/RTT/RTR, TTR/TRT/TRR/RTT/RRT, TTR/TRT/TRR/RTT/RRR, TTR/TRT/TRR/RTR/RRT, TTR/TRT/TRR/RTR/RRR, TTR/TRT/TRR/RRT/RRR, TTR/TRT/RTT/RTR/RRT, TTR/TRT/RTT/RTR/RRR, TTR/TRT/RTT/RRT/RRR, TTR/TRT/RTR/RRT/RRR, TTR/TRR/RTT/RTR/RRT, TTR/TRR/RTT/RTR/RRR, TTR/TRR/RTT/RRT/RRR, TTR/TRR/RTR/RRT/RRR, TTR/RTT/RTR/RRT/RRR, TRT/TRR/RTT/RTR/RRT, TRT/TRR/RTT/RTR/RRR, TRT/TRR/RTT/RRT/RRR, TRT/TRR/RTR/RRT/RRR, TRT/RTT/RTR/RRT/RRR, TRR/RTT/RTR/RRT/RRR 7-sequence (8 designs, 7 with non-switching arm): TTT/TTR/TRT/TRR/RTT/RTR/RRT, TTT/TTR/TRT/TRR/RTT/RTR/RRR, TTT/TTR/TRT/TRR/RTT/RRT/RRR, TTT/TTR/TRT/TRR/RTR/RRT/RRR, TTT/TTR/TRT/RTT/RTR/RRT/RRR, TTT/TTR/TRR/RTT/RTR/RRT/RRR, TTT/TRT/TRR/RTT/RTR/RRT/RRR, TTR/TRT/TRR/RTT/RTR/RRT/RRR

**Table A2:** Summary table of 4-period designs by three structures of treatment regimen.

4-Period Design (65,519 Designs)
**SBU Designs (1 design), including 1 NSA design** 4-sequence (1 design, 1 with non-switching arm) **SBUwP Designs (120 designs), including 60 NSA designs** 4-sequence (21 designs, 3 with non-switching arm) 8-sequence (76 designs, 38 with non-switching arm) 12-sequence (22 designs, 18 with non-switching arm) 16-sequence (1 design with non-switching arm)
**SB Designs (296 designs), including 148 NSA designs** 4-sequence (38 designs, 4 with non-switching arm) 8-sequence (220 designs, 110 with non-switching arm) 12-sequence (38 designs, 34 with non-switching arm) **UwP Designs (520 designs), including 264 NSA designs** 2-sequence (5 designs, 1 with non-switching arm) 4-sequence (28 designs, 9 with non-switching arm) 6-sequence (151 designs, 57 with non-switching arm) 8-sequence (146 designs, 73 with non-switching arm) 10-sequence (152 designs, 95 with non-switching arm) 12-sequence (30 designs, 21 with non-switching arm) 14-sequence (8 designs, 8 with non-switching arm) **Uniform Design(6 designs), including 0 NSA designs** 2-sequence (3 designs, 0 with non-switching arm) 4-sequence (2 designs, 0 with non-switching arm) 6-sequence (1 design, 0 with non-switching arm) **UwS Design(50 designs), including 0 NSA designs** 2-sequence (12 designs, 0 with non-switching arm) 3-sequence (20 designs, 0 with non-switching arm) 4-sequence (12 designs, 0 with non-switching arm) 5-sequence (6 designs, 0 with non-switching arm)
**None Designs (64,526 designs), including 32,295 NSA designs** 2-sequence (100 designs, 14 with non-switching arm) 3-sequence (540 designs, 105 with non-switching arm) 4-sequence (1718 designs, 438 with non-switching arm) 5-sequence (4362 designs, 1365 with non-switching arm) 6-sequence (7856 designs, 2946 with non-switching arm) 7-sequence (11,440 designs, 5005 with non-switching arm) 8-sequence (12,428 designs, 6214 with non-switching arm) 9-sequence (11,440 designs, 6435 with non-switching arm) 10-sequence (7856 designs, 4910 with non-switching arm) 11-sequence (4368 designs, 3003 with non-switching arm) 12-sequence (1730 designs, 1292 with non-switching arm) 13-sequence (560 designs, 455 with non-switching arm) 14-sequence (112 designs, 98 with non-switching arm) 15-sequence (16 designs, 15 with non-switching arm)

**Table A3:** Four-period SBUwP-NSA designs involving a non-switching arm that achieve the minimum variance for both with and without carryover effect scenarios.

4-Period SBUwP-NSA Design (61 Designs)
4 × 4 (4 designs): TTTT/TRTR/RTRT/RRRR, TTTR/TRTT/RTRT/RRRR, TTRT/TRTT/RTTR/RRRR, TTRT/TRTR/RTTT/RRRR
8 × 4 (38 designs): TTTT/TTTR/TTRT/TRTR/RTRT/RRTR/RRRT/RRRR, TTTT/TTTR/TRTT/TRTR/RTRT/RTRR/RRRT/RRRR, TTTT/TTTR/TRTT/TRRT/RTRT/RTRR/RRTR/RRRR, TTTT/TTTR/TRTR/TRRT/RTTR/RTRT/RRRT/RRRR, TTTT/TTTR/TRTR/TRRT/RTRT/RTRR/RRTT/RRRR, TTTT/TTRT/TTRR/TRTR/RTRT/RRTT/RRTR/RRRR, TTTT/TTRT/TRTT/TRTR/RTTR/RTRR/RRRT/RRRR, TTTT/TTRT/TRTT/TRRT/RTTR/RTRR/RRTR/RRRR, TTTT/TTRT/TRTT/TRRR/RTTR/RTRT/RRTR/RRRR, TTTT/TTRT/TRTR/TRRT/RTTT/RTRR/RRTR/RRRR, TTTT/TTRT/TRTR/TRRT/RTTR/RTRR/RRTT/RRRR, TTTT/TTRT/TRTR/TRRR/RTTT/RTRT/RRTR/RRRR, TTTT/TTRT/TRTR/TRRR/RTTR/RTRT/RRTT/RRRR, TTTT/TTRR/TRTT/TRTR/RTTR/RTRT/RRRT/RRRR, TTTT/TTRR/TRTT/TRTR/RTRT/RTRR/RRTT/RRRR, TTTT/TTRR/TRTT/TRRT/RTTR/RTRT/RRTR/RRRR, TTTT/TTRR/TRTR/TRRT/RTTT/RTRT/RRTR/RRRR, TTTT/TTRR/TRTR/TRRT/RTTR/RTRT/RRTT/RRRR, TTTT/TRTT/TRTR/TRRR/RTTT/RTRT/RTRR/RRRR, TTTT/TRTR/TRRT/TRRR/RTTT/RTTR/RTRT/RRRR, TTTR/TTRT/TTRR/TRTT/RTRT/RRTT/RRTR/RRRR, TTTR/TTRT/TRTT/TRTR/RTTT/RTRR/RRRT/RRRR, TTTR/TTRT/TRTT/TRRT/RTTT/RTRR/RRTR/RRRR, TTTR/TTRT/TRTT/TRRT/RTTR/RTRR/RRTT/RRRR, TTTR/TTRT/TRTT/TRRR/RTTT/RTRT/RRTR/RRRR, TTTR/TTRT/TRTT/TRRR/RTTR/RTRT/RRTT/RRRR, TTTR/TTRT/TRTR/TRRT/RTTT/RTTR/RRRT/RRRR, TTTR/TTRT/TRTR/TRRT/RTTT/RTRR/RRTT/RRRR, TTTR/TTRT/TRTR/TRRR/RTTT/RTRT/RRTT/RRRR, TTTR/TTRR/TRTT/TRTR/RTTT/RTRT/RRRT/RRRR, TTTR/TTRR/TRTT/TRRT/RTTT/RTRT/RRTR/RRRR, TTTR/TTRR/TRTT/TRRT/RTTR/RTRT/RRTT/RRRR, TTTR/TTRR/TRTR/TRRT/RTTT/RTRT/RRTT/RRRR, TTTR/TRTT/TRRT/TRRR/RTTT/RTTR/RTRT/RRRR, TTRT/TTRR/TRTT/TRTR/RTTT/RTTR/RRRT/RRRR, TTRT/TTRR/TRTT/TRTR/RTTT/RTRR/RRTT/RRRR, TTRT/TTRR/TRTT/TRRT/RTTT/RTTR/RRTR/RRRR, TTRT/TTRR/TRTR/TRRT/RTTT/RTTR/RRTT/RRRR,
12 × 4 (18 designs): TTTT/TTTR/TTRT/TTRR/TRTT/TRTR/RTRT/RTRR/RRTT/RRTR/RRRT/RRRR, TTTT/TTTR/TTRT/TTRR/TRTR/TRRT/RTTR/RTRT/RRTT/RRTR/RRRT/RRRR, TTTT/TTTR/TTRT/TRTT/TRTR/TRRR/RTTT/RTRT/RTRR/RRTR/RRRT/RRRR, TTTT/TTTR/TTRT/TRTT/TRTR/TRRR/RTTR/RTRT/RTRR/RRTT/RRRT/RRRR, TTTT/TTTR/TTRT/TRTT/TRRT/TRRR/RTTR/RTRT/RTRR/RRTT/RRTR/RRRR, TTTT/TTTR/TTRT/TRTR/TRRT/TRRR/RTTT/RTTR/RTRT/RRTR/RRRT/RRRR, TTTT/TTTR/TTRT/TRTR/TRRT/TRRR/RTTT/RTRT/RTRR/RRTT/RRTR/RRRR, TTTT/TTTR/TTRR/TRTT/TRTR/TRRT/RTTT/RTRT/RTRR/RRTR/RRRT/RRRR, TTTT/TTTR/TTRR/TRTT/TRTR/TRRT/RTTR/RTRT/RTRR/RRTT/RRRT/RRRR, TTTT/TTTR/TRTT/TRTR/TRRT/TRRR/RTTT/RTTR/RTRT/RTRR/RRRT/RRRR, TTTT/TTRT/TTRR/TRTT/TRTR/TRRT/RTTT/RTTR/RTRR/RRTR/RRRT/RRRR, TTTT/TTRT/TTRR/TRTT/TRTR/TRRR/RTTT/RTTR/RTRT/RRTR/RRRT/RRRR, TTTT/TTRT/TTRR/TRTT/TRTR/TRRR/RTTT/RTRT/RTRR/RRTT/RRTR/RRRR, TTTT/TTRT/TTRR/TRTR/TRRT/TRRR/RTTT/RTTR/RTRT/RRTT/RRTR/RRRR, TTTT/TTRR/TRTT/TRTR/TRRT/TRRR/RTTT/RTTR/RTRT/RTRR/RRTT/RRRR, TTTR/TTRT/TTRR/TRTT/TRTR/TRRT/RTTT/RTTR/RTRR/RRTT/RRRT/RRRR, TTTR/TTRT/TTRR/TRTT/TRTR/TRRR/RTTT/RTTR/RTRT/RRTT/RRRT/RRRR, TTTR/TTRT/TTRR/TRTT/TRRT/TRRR/RTTT/RTTR/RTRT/RRTT/RRTR/RRRR,
16 × 4 (1 design): TTTT/TTTR/TTRT/TTRR/TRTT/TRTR/TRRT/TRRR/RTTT/RTTR/RTRT/RTRR/RRTT/RRTR/RRRT/RRRR
